# Endoscopic Dissolution of Gastric Lipoma with Argon Plasma Coagulation

**DOI:** 10.7759/cureus.1526

**Published:** 2017-07-31

**Authors:** Abhilash Perisetti, Nayana George, Saikiran Raghavapuram, Abu Baker Sheikh, Mohit Girotra, Benjamin Tharian

**Affiliations:** 1 Department of Family and Community Medicine, Texas Tech University; 2 Department of Internal Medicine, University of Arkansas for Medical Sciences; 3 Division of Gastroenterology, University of Arkansas for Medical Sciences; 4 Student, Texas Tech University Health Sciences Center; 5 Department of Advanced Endoscopy, Stanford University Medical Center

**Keywords:** endoscopy, gastric lipoma, argon plasma coagulation

## Abstract

A 47-year-old patient presented from outside the hospital for evaluation of iron deficiency anemia (IDA). The endoscopic workup suggested a gastric antral subepithelial lesion with an overlying arteriovenous malformation (AVM). Endoscopic ultrasound (EUS) revealed the lesion to be a lipoma. Given the patient’s anemia and blood transfusion requirements, the AVM was treated with argon plasma coagulation (APC). During this treatment, desiccation of fat was noted with a significant decrease in the size of the subepithelial lesion (the gastric lipoma). While the APC therapy was intended for management of the overlying AVM, it resulted in the partial dissolution of the gastric lipoma, proving to be a potential diagnostic and therapeutic tool.

## Introduction

Lipomas constitute about 3% of gastrointestinal tumors with a predominant location in the colon (60-75%) and the small intestine (30%) [1]. The occurrence of lipomas in the stomach is rare (5%). They are mostly asymptomatic and incidental in nature. Some lipomas may become symptomatic and can result in gastrointestinal bleeding, iron-deficiency anemia, gastric outlet obstruction, and intussusceptions [2-5]. Endoscopy reveals a classic yellowish, smooth subepithelial mass, with frequently styled features like tenting, cushion sign, or naked fat sign [1]. Endoscopic ultrasound (EUS) is highly sensitive in characterizing lipomas as hyperechoic lesions arising from the submucosal layer. Symptomatic lesions are surgically managed with laparoscopic or open resection, or endoscopic submucosal dissection [1]. We report a case of a gastric lipoma with a large overlying arteriovenous malformation (AVM) causing blood loss anemia. Argon plasma coagulation (APC) treatment was planned for the AVM. During treatment for the AVM, fat was seen desiccating from the underlying gastric lipoma. Repeat EUS demonstrated a significant decrease in the size of the lipoma. APC has been reported for resection of airway lipomas. However, its use for gastric lipomas is rare [6]. APC of a lipoma could be a potential diagnostic and therapeutic tool, especially if it co-exists with a gastric lipoma. Endoscopic or surgical resection is preferred if an AVM co-exists with a subepithelial lesion, other than a lipoma. To our knowledge, this is the first case of the use of APC for resection of a gastric lipoma co-existing with an AVM.

## Case presentation

A 47-year-old man was referred to our facility for evaluation and management of iron deficiency anemia (IDA). He had no history of melena, hematemesis, or bright red blood per rectum. He denied any recent diarrhea and intolerance to gluten. No family history of celiac disease or colon cancer was reported. There was no history of shortness of breath or chest pain. He was recently started on iron supplements. There was no report of non-steroidal anti-inflammatory drugs (NSAID) use, alcohol or any illicit drugs. He denied a history of previous abdominal surgeries and a history of bleeding diathesis. On physical exam, this middle-aged Caucasian male appeared very pale. Oxygen saturation on pulse oximetry was 97%, blood pressure (BP) was 112/75, heart rate was 82/min, and temperature was 98.4 degrees Fahrenheit. He was alert and oriented times three.  Conjunctival pallor was noted. Pupils were about 4 millimeters (mm) bilaterally and reactive to light. On auscultation, he had dual heart sounds with no murmurs noted. Breath sounds were bilaterally equal with no crackles or rhonchi. The abdomen was soft, non-distended with no masses noted. On laboratory testing, his white blood cell count (WBC) was within normal limits at 9.6 g/dL, a hemoglobin of 9.5 g/dL, and a platelet count of 162,000. Electrolytes were within normal limits. He had an endoscopic workup done outside the hospital before being referred to us. Upper endoscopy (EGD) performed at the outside facility showed a gastric antral subepithelial lesion with overlying erosions, which were considered to be the source of his occult bleeding.  Gastric biopsies were negative for heliobacterium pylori and showed features of chronic gastritis with no features of atrophy or intestinal metaplasia. The colonoscopy and capsule endoscopy were reported as normal from the same facility.

**Figure 1:**
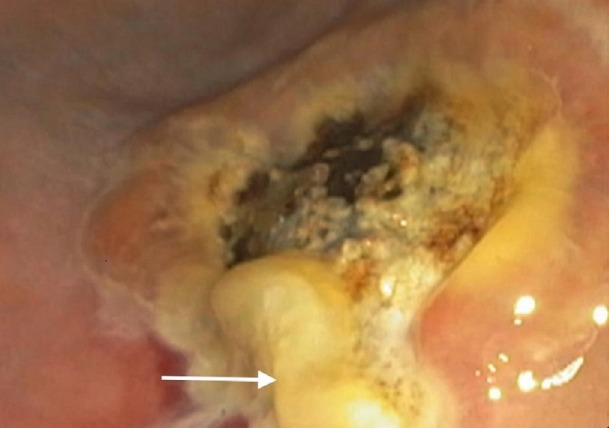
Fat noticed desiccating from the lipoma (arrow) after APC treatment

**Figure 2:**
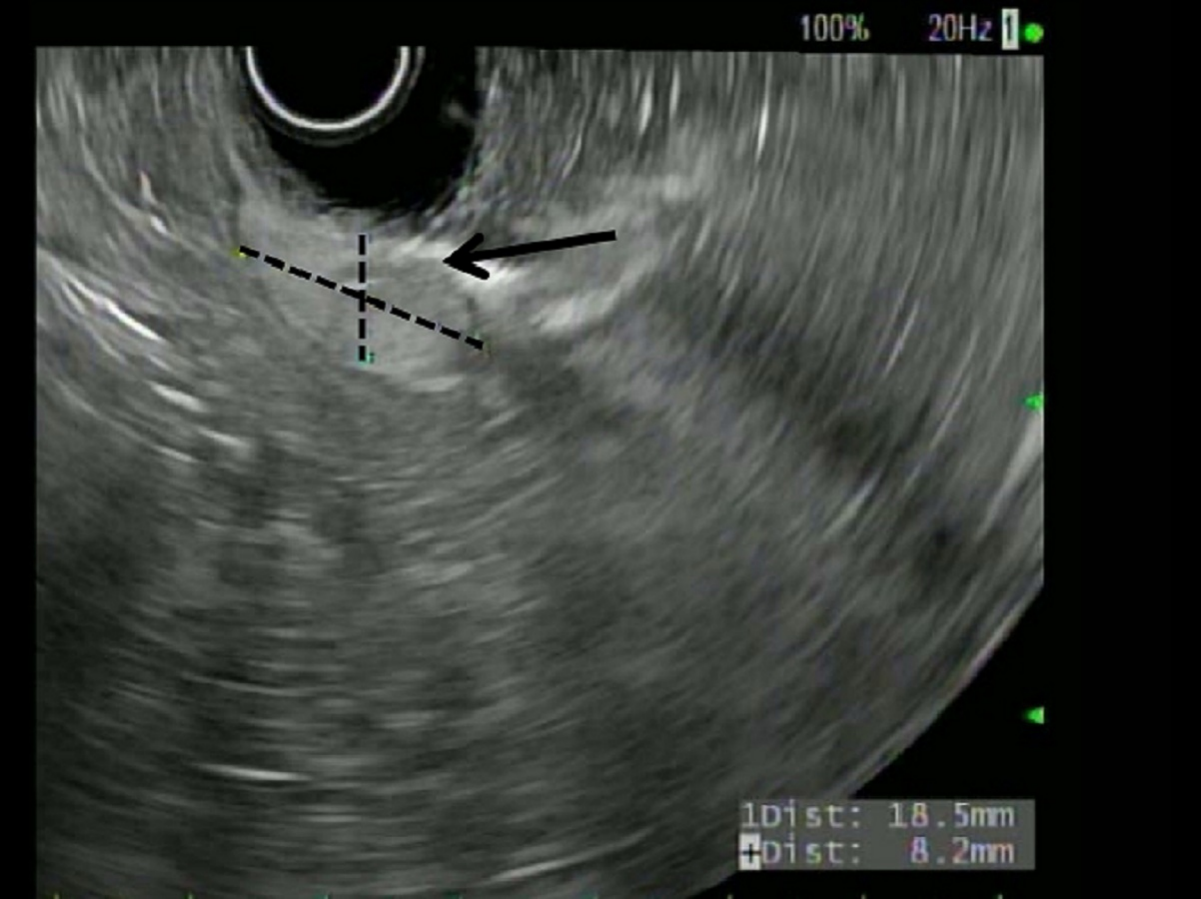
Endoscopic ultrasound showing a reduction in size of the lipoma after argon plasma coagulation (dotted lines)

Video 1 shows the endoscopy performed at our facility, which shows an approximately 4 cm x 3 cm subepithelial oval-shaped smooth nodule noted in the antrum, with a slight yellowish tint underneath, suggestive of a lipoma with an overlying 1.5 cm circular non-bleeding arteriovenous malformation (AVM) most prominent on the distal part of the nodule. The distal margins of the nodule appeared more defined, while the proximal margin tended to fade away. Distal to the nodule, there was a tattoo that had been placed outside of the hospital. Though not actively bleeding at the time of endoscopy, this AVM was deemed to be the plausible cause of his iron deficiency anemia through intermittent bleeding. Endoscopic ultrasound (EUS) showed a hyperechoic homogeneous lesion confined to the submucosa consistent with a lipoma. To control the bleeding source, argon plasma coagulation (APC) was used. Post-treatment, golden-yellow fat was seen extruding through the lesion with a significant decrease in the size of the lipoma (Figure 1). A repeat EUS showed a significant decrease in the size of the lipoma (Figure 2).

**Video 1:** Endoscopic findings suggestive of a lipoma

## Discussion

To our knowledge, this is the first case describing the simultaneous treatment of a lipoma and an AVM with APC in the gastrointestinal tract. Endoscopically, gastric lipomas are seen as subepithelial lesions among other causes, such as gastrointestinal stromal tumors (GIST) and leiomyomas [7]. They have characteristic features (tenting, cushion or naked fat sign) on endoscopic ultrasound (EUS), which help us to differentiate them from other etiologies [8]. They are asymptomatic in the majority of cases and are found incidentally. They do not need specific management unless they are symptomatic. Lipomas can be endoscopically resected by loop ligation, hybrid endoscopic submucosal dissection (ESD), or by the STER technique (submucosal tunneling and endoscopic resection) [9].

Argon plasma coagulation (APC) has been available in flexible endoscopy since 1991 and used in the treatment of various conditions, such as hemorrhage, malignant and benign tumors, tissue ingrowth, overgrowth of stents, and angiodysplasia [6]. The superficial location of angiodysplasias makes them well suited for APC treatment. Immediate hemostasis rates range from 85 - 100% in various reports with long-term control of bleeding [7]. In our case, APC was originally intended for the treatment of an overlying AVM, although it simultaneously caused regression of the co-existing lipoma (Figures 1-2). The application of APC for gastric lipomas could potentially be diagnostic and curative.

Though this case showed resolution of the lipoma due to the application of APC, there are potential limitations. Due to the thermal nature of APC, aggressive application for lipomas could potentially lead to deep thermal injury to walls and perforation [10]. The use of a touchless application and vigorous suctioning post-procedure could minimize excessive thermal injury and perforation. Endoscopic or surgical resection of gastric lipomas, though more invasive, is superior to APC when feasible in symptomatic gastrointestinal lipomas, with a more controlled setting and the advantage of histological diagnosis [9]. Though APC has limitations due to its thermal nature, it could potentially be used to decrease the size of lipomas, especially when they coexist with AVM and in poor surgical candidates who cannot tolerate endoscopic or surgical resection.

## Conclusions

Through this case, we demonstrate that APC could be used in the treatment of AVMs and gastric lipomas when they co-exist. APC could be used as a possible diagnostic and therapeutic adjunctive management strategy for symptomatic gastric lipomas when endoscopic removal is not feasible. The risk of thermal injury and potential perforation is a possibility. Debulking with APC can also be a considered in symptomatic lipomas without co-existing AVM's in high-risk surgical candidates, although future studies are needed to confirm this.
